# Limited resection and two-staged lobectomy for non-small cell lung cancer with ground-glass opacity

**DOI:** 10.1186/1749-8090-8-111

**Published:** 2013-04-24

**Authors:** Kazuto Ohtaka, Yasuhiro Hida, Kichizo Kaga, Tatsuya Kato, Jun Muto, Reiko Nakada-Kubota, Satoshi Hirano, Yoshiro Matsui

**Affiliations:** 1 Department of Cardiovascular and Thoracic Surgery, Hokkaido University Graduate School of Medicine, Sapporo, Hokkaido, Japan; 2 Gastroenterology Surgery II, Hokkaido University Graduate School of Medicine, Sapporo, Hokkaido, Japan; 3 Department of Cardiovascular and Thoracic Surgery, Hokkaido University Graduate School of Medicine, North 15, West 7, Kita-ku, Sapporo, Hokkaido 060-8638, Japan

**Keywords:** Ground-glass opacity, Two-staged surgery, Noguchi type, Lung cancer, Adenocarcinoma

## Abstract

**Background:**

Lung tumors showing ground-glass opacities on high-resolution computed tomography indicate the presence of inflammation, atypical adenomatous hyperplasia, or localized bronchioloalveolar carcinoma. We adopted a two-staged video-assisted thoracoscopic lobectomy strategy involving completion lobectomy for localized bronchioloalveolar carcinoma with an invasive component according to postoperative pathological examination by permanent section after partial resection.

**Methods:**

Forty-one patients with undiagnosed small peripheral ground-glass opacity lesions underwent partial resection from 2001 to 2007 in Hokkaido University Hospital. Localized bronchioloalveolar carcinoma was classified according to the Noguchi classification for adenocarcinoma. Malignant lesions other than Noguchi types A and B were considered for completion lobectomy and systemic mediastinal lymphadenectomy. Perioperative data of completion video-assisted thoracoscopic lobectomies were compared with data of 67 upfront video-assisted thoracoscopic lobectomies for clinical stage IA adenocarcinoma performed during the same period.

**Results:**

Postoperative pathological examination revealed 35 malignant and 6 non-malignant diseases. Histologically, all of the malignant diseases were adenocarcinomas of Noguchi type A (n = 7), B (n = 9), C (n = 18), and F (n = 1). Eleven of 19 patients (58%) with Noguchi type C or F underwent two-staged video-assisted thoracoscopic lobectomy. Three patients refused a second surgery. There was no cancer recurrence. The two-staged lobectomy group had a significantly longer operative time and more blood loss than the upfront lobectomy group. There was no surgical mortality or cancer recurrence.

**Conclusions:**

Two-staged lobectomy for undiagnosed small peripheral ground-glass opacity lesions showed satisfactory oncological results. However, low compliance for and invasiveness of the second surgery are concerns associated with this strategy.

## Background

In 1995, Noguchi et al. proposed the classification of adenocarcinoma into six pathologic types, A to F, based on growth patterns [1]. Noguchi type A adenocarcinoma is localized bronchioloalveolar carcinoma (LBAC), and Noguchi type B adenocarcinoma is LBAC with foci of structural alveolar collapse. Types A and B are not invasive, show no lymph node metastasis, and have a 5-year survival rate of 100%. Noguchi type C adenocarcinoma, which is LBAC with foci of active fibroblastic proliferation, has the potential for lymph node metastasis and has a 5-year survival rate of 75%. Noguchi type was subsequently reported to be associated with radiological findings on high-resolution computed tomography (HRCT) imaging [2-4]. Most cases of bronchioloalveolar carcinoma (BAC), such as Noguchi types A, B, and C, have radiological findings of ground-glass opacities (GGO) on HRCT. Aoki et al. reported that most Noguchi type A or B adenocarcinomas showed localized GGO on HRCT, and that most Noguchi type C adenocarcinomas showed partial GGO mixed with localized solid attenuation [2]. Kondo et al. reported that 81.8% of air-containing tumors with less than 50% opacity on mediastinal window images were Noguchi type A or B, and 88.7% of the solid-density tumors with more than 50% opacity were Noguchi type C, D, or F [3]. Kuriyama et al. reported that the percentage of GGO in LBAC was significantly greater than that in adenocarcinoma with or without a replacement growth pattern [4]. That is, tumors that comprise a larger GGO component on HRCT, especially tumors with pure GGO, are likely to be Noguchi type A or B adenocarcinoma.

Given that tumors demonstrating pure GGO or mixed GGO lesions with a slight solid part represent early-stage cancer without invasiveness, they might be good candidates for limited surgery with curative intent. The outcome of limited surgery with curative intent for patients with pure GGO lesions is reportedly satisfactory [5-7]. Although BAC generally has a good prognosis, some cases, especially type C, have lymph node involvement and recurrence [8]. Some reports have investigated whether limited resection or lobectomy should be performed for GGO lesions according to the intraoperative pathological diagnosis of Noguchi type based on frozen section of partially resected specimens [9-12]. That is, if the tumor is diagnosed as Noguchi type A or B adenocarcinoma or noninvasive carcinoma based on intraoperative pathological examination with frozen section after partial resection, then additional resection is not indicated unless the surgical margin is positive. If the tumor is diagnosed as another Noguchi type of adenocarcinoma or invasive carcinoma, lobectomy with mediastinal lymph node dissection is performed successively. The above-mentioned studies reported that the results of this therapeutic strategy are satisfactory in terms of patient survival.

However, there was a limitation in this therapeutic strategy: intraoperative pathological examination sometimes led to a misdiagnosis of Noguchi type or cancer invasiveness, because the Noguchi type of adenocarcinoma was originally diagnosed based on postoperative pathological examination by permanent section [5,10,13]. Misdiagnosis of Noguchi type would result in either over- or undertreatment in the form of lobectomy for patients with noninvasive cancer or insufficient limited surgery for patients with invasive cancer, respectively.

To avoid these intraoperative pathologic diagnostic errors and resultant inappropriate surgeries, we adopted the following therapeutic strategy for patients with undiagnosed small peripheral GGO lesions for 7 years (2001–2007). According to the pathological diagnosis by permanent section after partial resection, patients with Noguchi type A or B adenocarcinoma underwent postoperative follow-up without two-staged lobectomy. For other Noguchi type adenocarcinomas (including type C) and cancers of other histological types, two-staged lobectomy was considered. There have been no reports of a therapeutic strategy similar to ours. We herein report the perioperative and survival outcomes of this two-staged lobectomy strategy based on pathological examination by permanent section in patients with undiagnosed small peripheral GGO lesions.

## Patients and methods

The therapeutic strategy for patients with undiagnosed small peripheral GGO lesions in our institute is as following. First, video-assisted thoracoscopic (VATS) partial resection was performed after obtaining informed consent for the possibility of two-staged lobectomy. Based on postoperative pathological examination by permanent section, patients who were diagnosed with non-malignant disease or Noguchi type A or B adenocarcinoma did not undergo two-staged lobectomy. On the other hand, patients who were diagnosed with adenocarcinoma other than Noguchi type A or B or with cancers of other histologic types underwent two-staged lobectomy if the patient met the medical criteria for lobectomy and agreed to the second surgery. The institutional review board of Hokkaido university hospital approved this retrospective study and waived the requirement for informed consent*.*

**Figure 1:**
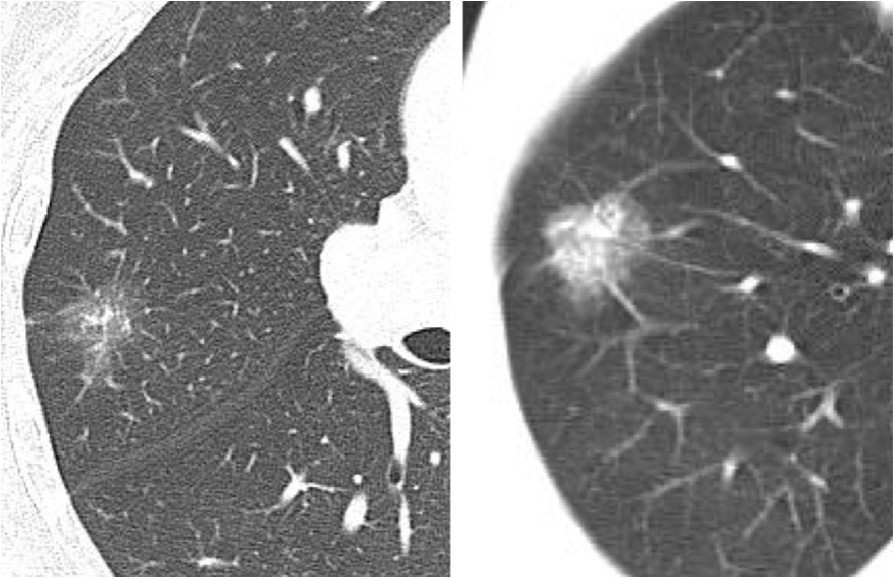
CT findings of pure (left) and mixed (right) ground-glass opacity lesions.

The subjects were 41 patients with undiagnosed small peripheral GGO lesions who underwent pulmonary resection in Hokkaido University Hospital from January 2001 to December 2007. The clinicopathological data were obtained from the medical charts. GGO was defined as a localized or focal misty increase in lung attenuation that did not obscure the underlying vascular markings. Typical CT findings of GGO lesions were shown in Figure 1. The peripheral lung field was defined as the outer one-third of lung field on CT imaging. For unpalpable tumors, gold markers were preoperatively inserted to identify the location intraoperatively. Details on the technique for inserting the 1.5-mm-diameter gold markers into the lung have been previously reported [14]. In brief, special equipment that was developed for the insertion of gold markers using bronchoscopy was used to insert the markers into small bronchi (Olympus, Tokyo, Japan).

The usefulness and safety of our therapeutic strategy for patients with undiagnosed small peripheral GGO lesions were statistically analyzed. To evaluate usefulness, overall survival (OS) and DFS after resection were analyzed. OS was the period between the date of operation and the date of death or the last date of confirmed survival. DFS was the period between the date of operation and the date of recurrence or the last date of confirmed recurrence-free survival. For patients who underwent two-staged lobectomy, OS and DFS were the period between the date of first operation and the last date.

To evaluate safety, perioperative data of patients who underwent two-staged VATS lobectomy (two-staged lobectomy group), such as operative time, blood loss, ratio of conversion to open thoracotomy from VATS lobectomy, postoperative complications, period of postoperative drainage, length of postoperative hospital stay, and surgery-related death, were compared with data of 67 patients with adenocarcinoma who were diagnosed with clinical stage IA disease and underwent VATS lobectomy during the same period (upfront lobectomy group). The following patients were excluded: patients who underwent partial resection of another lobe at the same time and patients with a past history of lung resection. Conversion to open thoracotomy from VATS lobectomy was performed if there was severe adhesion of lymph nodes to the pulmonary artery, severe adhesion of the pleura, or uncontrolled vascular injury. Postoperative pulmonary fistula was defined as air leakage that continued for more than 7 postoperative days or necessitated pleurodesis or reoperation.

The survival rate was calculated using the Kaplan-Meier method. Comparison between the two-staged and upfront lobectomy groups was performed using the Mann–Whitney U test and chi-square test. All statistical analyses were performed using StatView 5.0 software (SAS Institute Inc., Cary, NC, USA). A p-value of <0.05 indicated a significant difference.

## Results

**Figure 2:**
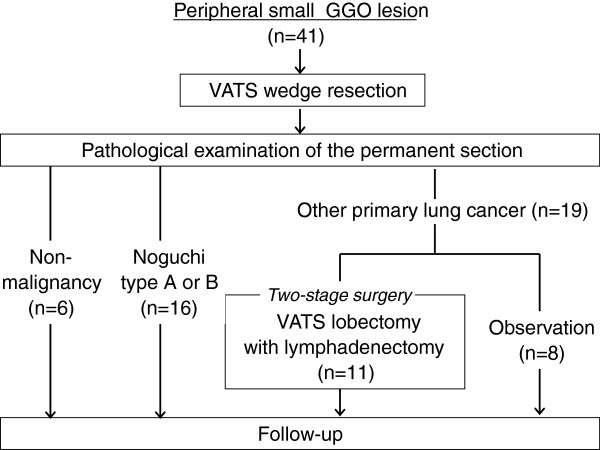
Patient breakout in the present study.

The characteristics of 41 patients with preoperatively undiagnosed small peripheral GGO lesions are shown in Table 1. The 41 patients comprised 16 males and 25 females with a median age of 63 years (range, 37–79 years) and a median tumor size on CT of 13 mm (range, 5–20 mm). The tumor location was the right upper lobe in 24 patients, left lower lobe in 8, left upper lobe in 7, right middle lobe in 1, and right lower lobe in 1. The postoperative pathological examination by permanent section resulted in a pathological diagnosis of 35 malignant and 6 non-malignant diseases (4 atypical adenomatous hyperplasia, a intrapulmonary bleeding and an inflammatory lesion). Histologically, all of the malignant diseases were adenocarcinomas of Noguchi type A (n = 7), B (n = 9), C (n = 18), and F (n = 1). Eight of 19 patients (42%) with Noguchi type C and F did not undergo two-staged lobectomy for the following reasons: preexisting diseases such as intestinal pneumonia in 3 patients, patient refusal in 3, and multiple lesions in 2. Finally, 11 patients (58%) underwent two-staged VATS lobectomy with systemic lymph node dissection (Figure 2). The median period between partial resection and two-staged lobectomy was 44 days (range, 14–248 days).

**Table 1:** Patient characteristics

**Characteristics**		**The patients with GGO (n = 41)**
Age (year)		
Median value (range)		63 (37–79)
Sex		
Male		16 (39%)
Female		25 (61%)
CEA (ng/ml)		
Median value (range)		2.2 (0.5-18.8)
Location		
Right upper lobe		24 (58.5%)
Right middle lobe		1 (2.4%)
Right lower lobe		1 (2.4%)
Left upper lobe		7 (17.1%)
Left lower lobe		8 (19.5%)
Tumor size on CT (mm)		
Median value (range)		13 (5–20)
Histological type		
Adenocarcinoma		35 (85.4%)
Noguchi type		
	A	7
	B	9
	C	18
	F	1
Non-malignancy		6 (14.6%)
Tumor size on specimen (mm)		
Median value (range)		13 (5–22)
TNM classification		
T1a N0 M0		33
T1b N0 M0		2

**Figure 3:**
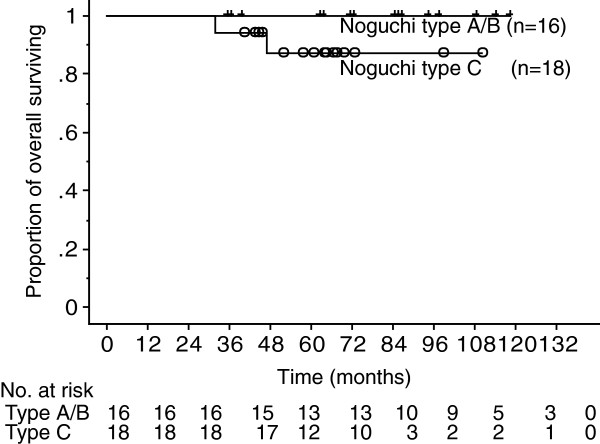
Overall survival curve of patients with undiagnosed small peripheral ground-glass opacity lesions using our therapeutic strategy based on postoperative pathological examination by permanent section.

The median postoperative follow-up period was 67 months (range, 32–119 months) in 41 patients with GGO lesions and 67 months (range, 32–119 months) in 35 patients with malignant diseases. Although two patients with Noguchi type C adenocarcinoma died of other diseases, no patents had recurrence of lung cancer. In patients with Noguchi type A or B adenocarcinoma, the 5-year OS and DFS were 100% (Figure 3). In patients with Noguchi type C adenocarcinoma, the 5-year OS was 87.2% and the 5-year DFS was 100%.

Patient characteristics of the two-staged (n = 11) and upfront lobectomy (n = 67) groups are shown in Table 2. The tumor size of the two-staged lobectomy group was significantly smaller than that of the upfront group (p = 0.0138). None of the other factors were significantly different. To evaluate safety, perioperative data of the two-staged group were compared with those of the upfront group (Table 3). The two-staged group had a median operative time of 306 minutes (range, 199–499 minutes), which was significantly longer than that of the upfront group, which had a median value of 250 minutes (range, 141–469 minutes) (p = 0.0069). The two-staged group had a median blood loss of 210 ml (range, 20–550 ml), which was significantly more than that of the upfront group, which had a median value of 80 ml (range, 0–1050 ml) (p = 0.0138). The ratio of conversion to open thoracotomy in the two-staged group was higher (18.2%), but the difference was not significant. There was no significant difference in other factors between the two groups.

**Table 2:** Patient characteristics in two-staged and upfront lobectomy groups

**Characteristics**	**Two-staged lobectomy group (n = 11)**	**Upfront lobectomy group (n = 67)**	**p value**
Age (y.o.)			
Median value (range)	67 (45–75)	66 (31–86)	0.446
Sex			
Male	5 (45.5%)	30 (44.8%)	>0.999
Female	6 (54.5%)	37 (55.2%)	
CEA (ng/ml)			
Median value (range)	2.4 (1.4-18.8)	2.7 (5.0-95.0)	0.807
Location			
Right upper lobe	6 (54.5%)	29 (43.3%)	0.524
Right middle lobe	1 (9.1%)	7 (10.4%)	
Right lower lobe	0 (0%)	13 (19.4%)	
Left upper lobe	2 (18.2%)	12 (17.9%)	
Left lower lobe	2 (18.2%)	6 (9.0%)	
Tumor size on CT (mm)			
Median value (range)	13 (9–20)	20 (7–30)	0.002
Tumor size on specimen (mm)			
Median value (range)	15 (8–20)	18 (6–63)	0.014
TNM classification			
T1a / T1b / T2a / T2b / T3	11 / 0 / 0 / 0 / 0	35 / 18 / 8 / 1 / 5	0.063
N0 / N1 / N2	11 / 0 / 0	60 / 3 / 4	0.532
Stage IA / IB / IIA / IIB / IIIA	11 / 0 / 0 / 0 / 0	47 / 8 / 4 / 4 / 4	0.353

**Table 3:** Perioperative data in two-staged and upfront lobectomy groups

**Characteristics**	**Two-staged lobectomy group (n = 11)**	**Upfront lobectomy group (n = 67)**	**p-value**
Conversion to open thoracotomy			
	2 (18.2%)	5 (7.5%)	0.255
The reasons of conversion			
Strong adhesion of lymph node	0	3	
Pleural adhesion	2	2	
Operative time (min.)			
Median value (range)	306 (199–499)	250 (141–469)	0.007
Blood loss (ml)			
Median value (range)	210 (20–550)	80 (0–1050)	0.014
Complication			
	3 (27.3%)	12 (17.9%)	>0.999
Pulmonary fistula	1	4	
Chylothorax	2	1	
Empyema	0	1	
Arrhythmia	0	2	
Others	0	4	
Drainage period (day)			
Median value (range)	3 (1–14)	4 (1–13)	0.959
Postoperative hospital stay (day)			
Median value (range)	11 (7–27)	11 (5–27)	0.480
Operative death			
	0	0	-

## Discussion

In this study, we reported the results of limited resection for Noguchi type A and B BAC and two-staged lobectomy for other primary lung cancers. In patients with Noguchi type A or B adenocarcinoma (n = 16), the 5-year OS and DFS were 100%. In patients with Noguchi type C adenocarcinoma (n = 18), the 5-year OS and DFS were 87.2% and 100%, respectively. Patient survival was satisfactory and comparable with that of reports from other institutes. The postoperative follow-up period in the present study might not be long enough, because adenocarcinoma often recurs after 5 postoperative years [15,16].

There have been several reports on the therapeutic strategy in which partial resection is initially performed for tumors comprising a GGO component, and whether lobectomy was added was determined based on the intraoperative Noguchi type diagnosis on pathological examination by frozen section [10-12]. Lobectomy was not added after partial resection if the intraoperative pathological diagnosis was Noguchi type A or B, and lobectomy was added if another Noguchi type was diagnosed. Koike et al. reported that this therapeutic strategy resulted in a 5-year DFS of 93% and a 5-year disease-specific survival of 100% [10]. Yamato et al. reported that this therapeutic strategy resulted in no recurrence during 30 months of follow-up [11]. Ichiki et al. reported that this therapeutic strategy resulted in no recurrence and a 5-year OS of 100% in patients who were diagnosed with Noguchi A or B disease on intraoperative pathological examination by frozen section [12]. These reports demonstrated a good survival outcome with this strategy.

There is a limitation in Noguchi type diagnosis based on intraoperative pathological examination by frozen section. Determination of the Noguchi type of adenocarcinoma was originally based on postoperative pathological examination by permanent section [1]. Yoshida et al. reported a 98% diagnostic ratio on intraoperative pathological examination by frozen section [13]. One patient was diagnosed with Noguchi type B disease on intraoperative examination by frozen section, but converted to Noguchi type C on postoperative examination by permanent section. Koike et al. performed a prospective study in which limited resection was performed on 46 patients who were diagnosed with noninvasive BAC on intraoperative pathological examination by frozen section. Three patients were diagnosed with invasive adenocarcinoma on postoperative pathological examination by permanent section [10]. Yamada et al. reported that among 42 pure GGO lesions of 2 cm or less, 3 were diagnosed as Noguchi type C and 1 was diagnosed as Noguchi type A or B on intraoperative pathological examination by frozen section [5]. Ichiki et al. reported that of 16 patients who were diagnosed with Noguchi type A or B disease intraoperatively, 3 were diagnosed with Noguchi C disease postoperatively [12]. Yoshida et al. reported three recurrent patients who were diagnosed with Noguchi type B disease on intraoperative examination and underwent limited resection [17]. One had metachronous cancer, one had recurrence of the bronchial stump, and another had Noguchi type C disease on postoperative examination. Although whether lobectomy can prevent recurrence in patients with Noguchi C disease is unclear, a two-staged lobectomy strategy can avoid undertreatment.

We adopted a two-staged lobectomy strategy to avoid misdiagnosis due to intraoperative pathological examination by frozen section for undiagnosed small peripheral GGO lesions. That is, two-staged lobectomy was added based on the results of postoperative pathological examination by permanent section after partial resection. No patients who underwent this protocol were subjected to surgical overtreatment. However, of 19 patients with a diagnosis other than Noguchi type A or B disease, 8 (42%) did not undergo two-staged lobectomy. Although five of them did not undergo this procedure for medical reasons, three refused. There seems to be a problem with compliance to undergo a second surgery. It might be psychologically and financially overwhelming. The ratio of undertreatment was higher than the ratio of misdiagnosis based on intraoperative pathological examination in past reports [12].

Eleven patients underwent two-staged VATS lobectomy. The perioperative outcomes of these surgeries were compared with those of upfront VATS lobectomy for c-stage IA adenocarcinoma. Although there was no mortality in either group and no significant differences in morbidity, the time of operation and estimated blood loss were significantly greater in the two-staged group than in the upfront lobectomy group. The rate of conversion to open thoracotomy was as high as 18% in the two-staged group, although there was no significant difference with the upfront lobectomy group (7.5%, p = 0.255). It is speculated that two-staged lobectomy requires dissection of adhesions, and thus results in a longer operative time, more blood loss, and sometimes conversion to open thoracotomy. Two-staged lobectomy seems more invasive than upfront lobectomy. Recently, to avoid adverse effects of two-staged surgery, we performed limited resection according to the GGO ratio and standardized uptake value of PET-CT regardless of Noguchi classification with permanent section.

## Conclusions

The therapeutic strategy for undiagnosed small peripheral GGO lesions (i.e., two-staged lobectomy based on postoperative pathological examination by permanent section after partial resection) had satisfactory oncological results. However, there are concerns in terms of the invasiveness of the procedure and low compliance for two-staged lobectomy.

## Competing interest

The authors declare that they have no competing interests.

## Authors’ contributions

KO and YH wrote the manuscript. Other authors collected data. All authors read and approved the final manuscript.
